# Radiological Imaging Findings of Adrenal Abnormalities in TAFRO Syndrome: A Systematic Review

**DOI:** 10.3390/biomedicines12040837

**Published:** 2024-04-10

**Authors:** Ryo Kurokawa, Akira Baba, Rui Kano, Yo Kaneko, Mariko Kurokawa, Wataru Gonoi, Osamu Abe

**Affiliations:** 1 Department of Radiology, Graduate School of Medicine, The University of Tokyo, 7-3-1, Hongo, Bunkyo-ku, Tokyo 113-8655, Japan; kurokawam-rad@h.u-tokyo.ac.jp (M.K.); gonoiw-rad@h.u-tokyo.ac.jp (W.G.); abediag@g.ecc.u-tokyo.ac.jp (O.A.); 2 Department of Radiology, The Jikei University School of Medicine, 3-25-8, Nishi-Shimbashi, Minato-ku, Tokyo 105-8461, Japan; akirababa@jikei.ac.jp (A.B.); ruikano@jikei.ac.jp (R.K.); 3 Department of Radiology, Gifu University, 1-1 Yanagido, Gifu City 501-1194, Japan; kaneko.yo.t0@f.gifu-u.ac.jp

**Keywords:** TAFRO syndrome, adrenal gland, computed tomography, magnetic resonance imaging, multicentric Castleman disease

## Abstract

This systematic review article aims to investigate the clinical and radiological imaging characteristics of adrenal abnormalities in patients with thrombocytopenia, anasarca, fever, reticulin fibrosis, renal dysfunction, and organomegaly (TAFRO) syndrome. We searched the literature in PubMed, the Cochrane Library, and the Web of Science Core Collection. Ultimately, we analyzed 11 studies with 22 patients plus our 1 patient, totaling 23 patients. The mean age was 47.0 ± 12.6 years. There were 20 male and 3 female patients, respectively. The histopathological analysis of lymph nodes was conducted in 15 patients (65.2%), and the diagnosis was consistent with TAFRO syndrome in all 15 patients. Among the 23 patients, 11 patients (18 adrenal glands) showed adrenal ischemia/infarction, 9 patients (13 adrenal glands) showed adrenal hemorrhage, and 4 patients (7 adrenal glands) showed adrenomegaly without evidence of concurrent ischemia/infarction or hemorrhage. One patient demonstrated unilateral adrenal hemorrhage and contralateral adrenomegaly. In patients with adrenal ischemia/infarction, the adrenal glands displayed poor enhancement through contrast-enhanced computed tomography (CT). In patients with adrenal hemorrhage, the adrenal glands revealed high attenuation through non-enhanced CT and hematoma through magnetic resonance imaging. Adrenomegaly, with or without adrenal ischemia/infarction or hemorrhage, was observed in all patients (23/23, 100%). The subsequent calcification of the affected adrenal glands was frequently observed (9/14, 64.3%) when a follow-up CT was performed. Abdominal pain was frequent (15/23, 65.2%), all of which occurred after the disease’s onset, suggesting the importance of considering TAFRO syndrome as a cause of acute abdomen. Given the absence of evidence of adrenal abnormalities in non-TAFRO-idiopathic multicentric Castleman disease (iMCD), they may serve as diagnostic clues for differentiating TAFRO syndrome from non-TAFRO-iMCD.

## 1. Introduction

Thrombocytopenia, anasarca, fever, reticulin fibrosis, renal dysfunction, and organomegaly (TAFRO) syndrome is a systemic inflammatory disease that was first reported by Takai et al. in 2010 [1]. While some physicians consider it a variant of histologically similar HHV-8-unrelated (idiopathic) multicentric Castleman disease (iMCD-TAFRO) [2,3], others emphasize the differences between the two and affirm the existence of a TAFRO syndrome unrelated to iMCD. The latter group does not require the histological diagnosis of lymph nodes to diagnose TAFRO syndrome [4,5]. Regardless of the criteria, the exclusion of the following similar diseases is essential and commonly emphasized [2,5]: malignancies (lymphoma, cancer, etc.), infectious diseases, autoimmune/rheumatologic diseases, and POEMS syndrome. In terms of computed tomography (CT) imaging findings, Kiguchi et al. [6] reported that, in addition to anasarca (pleural effusion, ascites, pericardial effusion, periportal collar, gallbladder wall edema, subcutaneous edema, retroperitoneal edema, and mesenteric edema), and organomegaly (hepatosplenomegaly and lymphadenopathy), diffuse ground-glass bone lesions and “matted” anterior mediastinal lesions were frequently observed. Our group reported that in cases of TAFRO syndrome within three weeks of onset, anasarca (pleural effusion, ascites, and subcutaneous edema) was not observed in about half of the cases. Meanwhile, adrenal abnormalities (ischemia/infarction, hemorrhage, or adrenomegaly) were observed in 7 out of 13 cases (53.8%) over time [7]. Recent studies on adrenal abnormalities in TAFRO syndrome, namely adrenal ischemia/infarction, hemorrhage, and adrenomegaly, have been on the rise. These adrenal abnormalities are scarcely reported in non-TAFRO-iMCD, which has led to increased attention as potential distinguishing features between TAFRO syndrome and non-TAFRO-iMCD. In this systematic review article, we focus on the clinical and radiological findings of adrenal abnormalities in TAFRO syndrome.

## 2. Methods

### 2.1. Literature Search

To investigate studies of radiological findings of adrenal abnormalities in TAFRO syndrome, a search was conducted on 21 March 2024, using the following search terms, without any language or date limits:

- (TAFRO) AND (adrenal) on PubMed;
- TAFRO on the Cochrane Library;
- TAFRO AND adrenal on the Web of Science Core Collection.

Publications were considered eligible if they included all of the following criteria:

- The article includes patients with TAFRO syndrome with adrenal abnormalities, namely, adrenal ischemia/infarction, adrenal hemorrhage, and/or adrenomegaly.
- CT or MRI were performed.
- Each patient’s demographic and clinical information was available.

The exclusion criteria were as follows:

- CT or MRI images of the adrenal glands could not be evaluated.

Additionally, we performed another search on PubMed on 21 March 2024, using the following search terms: (“multicentric castleman”) AND (“adrenal”).

This study was performed according to the Preferred Reporting Items for Systematic Reviews and Meta-Analyses (PRISMA) 2020 statement [8]. The PROSPERO ID is CRD42024518890.

### 2.2. Criteria for TAFRO Syndrome

Iwaki’s criteria for diagnosing TAFRO-iMCD require the fulfilment of both histopathological criteria, i.e., (1) lymph node pathology findings compatible with TAFRO-iMCD and (2) negative tests for LANA-1 for HHV-8; of all major criteria, i.e., (i) the presence of three of five TAFRO symptoms, (thrombocytopenia, anasarca, fever, reticulin fibrosis, and organomegaly), (ii) the absence of hypergammaglobulinemia (immunoglobulin G < 3500 mg/dL), and (iii) small-volume lymphadenopathy; and of at least one minor criterion, i.e., (a) the hyper/normoplasia of megakaryocytes in the bone marrow and (b) high serum ALP levels without markedly elevated serum transaminase. Additionally, the following diseases must be excluded: rheumatologic diseases; infectious diseases; and neoplastic diseases, such as lymphoma, polyneuropathy, organomegaly, endocrinopathy, monoclonal gammopathy, skin changes (POEMS) syndrome, and other cancers [2]. 

Masaki’s updated criteria for diagnosing TAFRO syndrome in 2019 include the presence of all three major criteria, i.e., (1) anasarca; (2) thrombocytopenia, defined as a pre-treatment platelet count ≤ 100,000/mL without myelosuppressive treatment; and (3) systemic inflammation, defined as a fever of unknown etiology > 37.5 °C and/or a serum C-reactive protein concentration ≥ 2 mg/dL, and at least two of four minor criteria, i.e., (a) Castleman disease-like features on a lymph node biopsy, (b) reticulin myelofibrosis and/or an increased number of megakaryocytes in the bone marrow, (c) mild organomegaly, and (d) progressive renal insufficiency [5].

The main difference between Iwaki’s and Masaki’s updated diagnostic criteria is the presence or absence of lymph node pathology. Masaki’s criteria do not require the histopathological confirmation of lymph nodes for diagnosis, so physicians can initiate treatment without delay because sometimes a lymph node biopsy is impossible due to anasarca, bleeding tendencies, and/or the limited size of the targeted lymph node [5]. Conversely, this difference in diagnostic criteria is a potential source of heterogeneity among cases of TAFRO syndrome.

### 2.3. Data Analyses

Two board-certified diagnostic radiologists (with 9 and 12 years of experience in abdominal radiology, respectively) independently reviewed all studies and CT and MR images of the eligible cases. When discrepancies arose between the two reviewers, the ultimate decision was made by consensus.

### 2.4. Collected Data

The following data were collected.

Demographic data:

- Patient’s age;
- Sex;
- Histopathological diagnosis of lymph nodes (presence or absence).

Clinical data:

- Abdominal pain during the course of the disease (presence or absence);
- Abdominal pain at onset (presence or absence);
- Type of adrenal abnormalities (adrenal ischemia/infarction, adrenal hemorrhage, and/or adrenomegaly) within the articles of the study;
- Side of the affected adrenal gland (unilateral or bilateral).

Radiological data:

- Adrenal ischemia/infarction;
- Adrenal hemorrhage;
- Adrenomegaly;
- Adrenal calcification over time (presence or absence).

Adrenal ischemia/infarction was diagnosed when poorly enhanced areas were observed on contrast-enhanced study. Adrenal hemorrhage was diagnosed when high attenuation was observed through non-enhanced CT or hematoma was observed through MRI. Adrenomegaly was diagnosed subjectively with the consent of two radiologists. Subsequently, adrenal calcification was evaluated through a non-enhanced CT.

### 2.5. Quality Assessment

We employed a tool to evaluate the methodological quality of case reports and case series proposed by Murad et al. [9] This tool comprises eight signaling questions in four domains—selection, ascertainment, causality, and reporting—and has been used in many studies, including our previous ones [10,11,12,13].

## 3. Results

### 3.1. Study Selection

**Figure 1:**
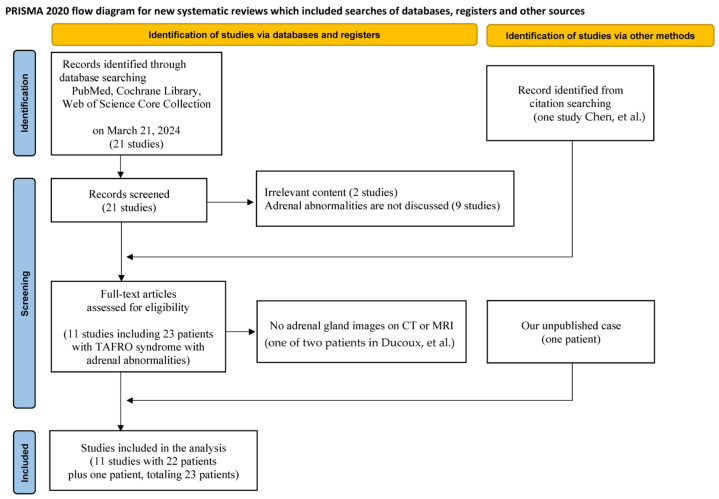
The flow diagram of study identification [19,23].

The first database search using PubMed, Cochrane Library, and Web of Science Core Collection identified 21 abstracts. Of these, 11 studies were excluded due to the following criteria: irrelevant content that did not include TAFRO syndrome (n = 2) and adrenal abnormalities were not discussed (n = 9). The remaining ten studies [7,14,15,16,17,18,19,20,21,22] were supplemented with one additional study [23] found outside the search list, totaling eleven studies, and our one unpublished case, culminating in an analysis of 24 patients. From these, one of the two patients in the study by Ducoux et al. [19] was excluded due to the absence of CT or MRI images of the adrenal glands. Ultimately, 23 patients were included in this study (Figure 1). The CT and MRI images of adrenal glands could be evaluated in 22 and 1 patient, respectively. The second database search yielded 22 hits, and of these, no study evaluated the relationship between adrenal abnormalities and non-TAFRO-iMCD.

### 3.2. Risk of Bias Assessment

As we extracted data from case-based studies in which the selection method was rarely mentioned, selection bias may have been introduced. The follow-up duration of the patients varied. Histopathological evaluations of the affected adrenal glands were performed in only 2 out of 23 cases [7,20].

### 3.3. Demographic and Clinical Data

The demographic and clinical data of the 23 patients are summarized in Table 1. The mean age was 47.0 ± 12.6 years. There were 20 male and 3 female patients, respectively. Histopathological analyses of lymph nodes were conducted in 15 cases, and in all patients, the diagnosis was consistent with TAFRO syndrome [7,14,15,16,17,19,20,21,22,23]. Ultimately, this study included eleven cases involving 18 adrenal glands showing adrenal ischemia/infarction, nine cases involving 15 adrenal glands showing adrenal hemorrhage, and four cases involving seven adrenal glands with adrenomegaly without evidence of adrenal ischemia or hemorrhage. In one case, unilateral adrenal hemorrhage and contralateral adrenomegaly were reported [15]. A follow-up CT scan was carried out in 14 patients approximately one month after the initial CT scan [7,15].

**Table 1:** Summary of patient data.

Study	Age [Year] Mean ± Standard Deviation	Sex	Histopathological Diagnosis of Lymph Node	Adrenal Findings	Unilateral or Bilateral	Concurrent Adrenomegaly in Ischemia/Infarction or Hemorrhage	Calcification Over Time	Abdominal Pain	Abdominal Pain at Disease Onset
	47.0 ± 12.6	Male = 20, Female = 3	YES = 15, NO = 8	Ischemia/infarction = 11, Hemorrhage = 9 *, Adrenomegaly without evidence of adrenal ischemia/infarction or hemorrhage = 4 *	Bilateral = 17, Unilateral = 6	YES = 20, NO = 0, Unknown = 0	YES = 9, NO = 5, Unknown = 9	YES = 15, NO = 8	YES = 15, NO = 8
Kurokawa [7]	24	Female	YES	Ischemia/infarction	Bilateral	YES	YES	YES	YES
	50	Male	NO	Ischemia/infarction	Unilateral	YES	NO	NO	NO
	71	Male	YES	Hemorrhage	Bilateral	YES **	NO	YES	YES
	33	Male	NO	Ischemia/infarction	Bilateral	YES	NO	YES	YES
	55	Male	NO	Ischemia/infarction	Bilateral	YES	NO	NO	NO
	53	Male	YES	Ischemia/infarction	Bilateral	YES	YES	NO	NO
	35	Male	YES	Adrenomegaly	Bilateral		NO	NO	NO
Fujimi [14]	54	Male	YES	Adrenomegaly	Bilateral		Unknown	YES	YES
Our case	38 (Figure 2)	Male	YES	Ischemia/infarction	Bilateral	YES	YES	YES	YES
Kano [15]	50 (Figure 3)	Male	NO	Hemorrhage and contralateral adrenomegaly *	Bilateral	YES	YES	YES	YES
	66	Male	YES	Hemorrhage	Bilateral	YES	YES	NO	NO
	45	Male	NO	Adrenomegaly	Bilateral		YES	NO	NO
	43	Male	NO	Hemorrhage	Bilateral	YES **	YES	YES	YES
	41	Male	NO	Hemorrhage	Bilateral	YES	YES	YES	YES
	51	Male	YES	Ischemia/infarction	Bilateral	YES	YES	YES	YES
Yonezaki [16]	53	Female	YES	Ischemia/infarction	Bilateral	YES	Unknown	YES	YES
Ono [17]	43	Male	YES	Ischemia/infarction	Unilateral	YES	Unknown	YES	YES
Okamoto [18]	70	Female	NO	Hemorrhage	Unilateral	YES	Unknown	YES	YES
Ducoux G [19]	19	Male	YES	Hemorrhage	Bilateral	YES	Unknown	YES	YES
Fujiwara [20]	46	Male	YES	Ischemia/infarction	Unilateral	YES	Unknown	YES	YES
Ito [21]	48	Male	YES	Hemorrhage	Bilateral	YES	Unknown	NO	NO
Nara [22]	48	Male	YES	Hemorrhage	Unilateral	YES	Unknown	NO	NO
Chen [23]	46	Male	YES	Ischemia/infarction	Unilateral	YES	Unknown	YES	YES

* In one patient, unilateral adrenal hemorrhage and contralateral adrenomegaly was observed. ** Concurrent adrenomegaly was observed in one of the two adrenal glands.

### 3.4. Adrenal Ischemia/Infarction

Adrenal ischemia or infarction in TAFRO syndrome was first reported by Fujiwara et al. [23]. We found 10 cases affecting 16 adrenal glands [7,15,16,17,20,23], and after adding our unpublished case involving bilateral adrenal glands, the total comes to 11 cases involving 18 adrenal glands with evaluable CT images.

This tally includes the report by Chen et al. [23] that described “adrenalitis”. Additionally, although the case reported by Ono et al. [17] was presented as bilateral, the contrast-enhanced CT scan in the figure depicts clear ischemic changes only on the left side; hence, it was counted as a unilateral case.

**Figure 2:**
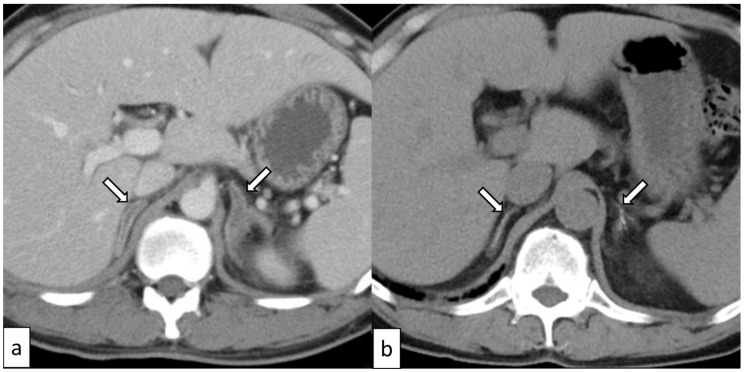
A 38-year-old man with TAFRO syndrome. A contrast-enhanced CT scan shows bilateral adrenomegaly and poorly enhanced adrenal glands ((**a**), arrows). One month later, the atrophy and calcification of the affected adrenal glands appear on a non-enhanced CT scan ((**b**), arrows).

Adrenal ischemia/infarction was observed on a contrast-enhanced CT scan as poorly enhanced areas of the adrenal glands, always accompanied by adrenomegaly, and the subsequent atrophy and calcification of the affected adrenal glands may be observed over time (Figure 2).

Clinically, eight out of eleven patients (72.7%) who presented with adrenal ischemia/infarction experienced abdominal pain during the course of the disease. All of the cases (8/8, 100%) reported abdominal pain from the onset, including the case outlined by Yonezaki et al. [16], where bilateral adrenal infarction was the initial presentation of TAFRO syndrome [7,16,17,20,23]. The only case of TAFRO syndrome with adrenal ischemia/infarction where adrenal pathology was obtained is the one case reported by Fujiwara et al. [20], which revealed necrosis and the formation of fibrotic granulomatous tissue without any evidence of epithelioid granuloma, lymphoma, or hemosiderin deposition.

### 3.5. Adrenal Hemorrhage

**Figure 3:**
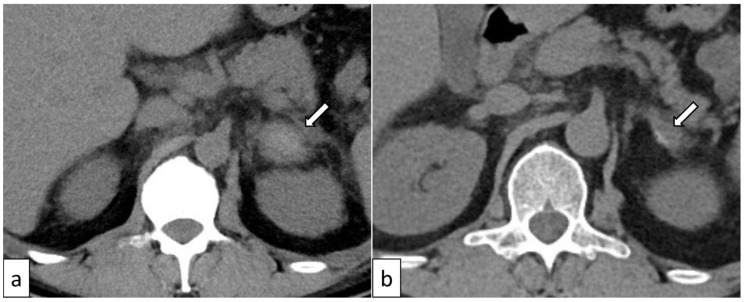
A 50-year-old man with TAFRO syndrome. A non-enhanced CT shows an enlarged and high-attenuated left adrenal gland, indicating adrenal hemorrhage ((**a**), arrow). Ten months later, the atrophy and calcification of the affected adrenal gland were observed ((**b**), arrow). CT images of the different parts of the body were evaluated in a previous study [15].

Adrenal hemorrhage in TAFRO syndrome was first reported by Nara et al. [22], and we found nine cases involving 15 adrenal glands with evaluable CT or MRI images (Table 1) [7,15,18,19,21,22]. Abdominal pain was experienced during the course of the disease in six out of nine cases (66.7%), with all of these (6/6, 100%) experiencing abdominal pain from the disease’s onset [7,14,15,18,19]. These data include information not mentioned in the article by Kano et al. [15]. Adrenal hemorrhage presents with simultaneous adrenomegaly in most patients (13/15 adrenal glands, 86.7%). CT scans usually show enlarged adrenal glands with high attenuation around 50–90 Hounsfield units (Figure 3). MR images were evaluated in only one case in the report by Ducoux et al. [19], and the affected adrenal glands showed hyperintensity on both T1- and T2-weighted images, indicating a subacute hemorrhage.

### 3.6. Adrenomegaly

Adrenomegaly without evidence of adrenal ischemia/infarction or adrenal hemorrhage was observed in four patients involving seven adrenal glands (Table 1) [7,14,15]. Contrast-enhanced CTs were performed in three of the four patients, while the other underwent non-enhanced CTs due to renal insufficiency. Abdominal pain was experienced in two patients (2/4, 50.0%) after the disease’s onset, including one patient with contralateral adrenal hemorrhage.

## 4. Discussion

In this systematic review, we evaluated the clinical and radiological findings of 23 patients with TAFRO syndrome presenting with adrenal abnormalities, namely, adrenal ischemia/infarction, adrenal hemorrhage, and/or adrenomegaly. Notably, most patients had abdominal pain (15/23, 65.2%) after the onset of the disease. The calcification of the adrenal glands over time was observed in 9 of 14 patients (64.3%) when a follow-up CT was evaluated approximately one month after the initial CT.

Adrenal abnormalities in TAFRO syndrome were first described by Finocchietto et al. [24], who noted “adrenal glands enlarged”, although CT or MR images of the adrenal glands were not available. Therefore, we did not include this study in this systematic review. Adrenal ischemia/infarction and adrenal hemorrhage were first described by Fujiwara et al. [20] and Nara et al. [22], respectively. In these three cases, histopathological analyses of the lymph nodes were conducted, and the results of the analysis were consistent with TAFRO syndrome. Anatomically, the adrenal glands are supplied by multiple arteries but have only one draining vein, which is why most non-TAFRO-related adrenal infarctions are known to occur venously, associated with infection, coagulability, and heart failure [25]. The mechanism of adrenal ischemia/infarction in TAFRO syndrome has not been specified. However, it may be caused by venous invasion by plasma cells or poor drainage due to para-neoplastic/para-viral autoimmunity. Moreover, we believe that lymphatic congestion related to the high affinity of TAFRO syndrome to the lymphatic system could also be a cause [7]. This point is discussed later.

The mechanism of adrenal hemorrhage in TAFRO syndrome, similar to ischemia/infarction, remains unclear, although it could be associated with venous or lymphatic congestion-induced hemorrhage, thrombocytopenia-related bleeding tendencies, or concurrent infections. Whether adrenal ischemia/infarction or hemorrhage, systemic hypercoagulability or embolic mechanisms seem unlikely because the simultaneous infarction or hemorrhage of other organs has not been demonstrated in cases of adrenal abnormalities in TAFRO syndrome. Concurrent infections may cause adrenal hemorrhage; however, in our previously reported autopsy case with adrenal hemorrhage, no evidence of infection was found [7]. Drug-induced adrenal hemorrhages, due to mediations such as corticosteroids, are unlikely because adrenal hemorrhages are often present before the diagnosis or treatment of TAFRO syndrome [26]. Adrenal hemorrhage is known to occur in malignant mimickers of TAFRO syndrome, namely, lymphomas and cancers [27,28]. However, as the histopathological evaluation of lymph nodes was performed in five out of nine cases (55.6%) with adrenal hemorrhages in TAFRO syndrome, and these malignancies are unlikely to be the cause of adrenal hemorrhage in TAFRO syndrome. Radiologically, nontraumatic adrenal hemorrhages are characterized by the evolution of a non-enhancing low- or mixed-attenuation mass in the involved adrenal glands [29]. High-intensity on T1-weighted imaging may be observed in MRI scans [29]. These imaging characteristics corroborated those of patients with TAFRO syndrome. Furthermore, it has been reported that in nontraumatic adrenal hemorrhage, bleeding often continues until the adrenal gland expands beyond the adreniform shape, resulting in a round or oval hematoma [29]. This explains the mechanism of the high incidence of adrenomegaly in patients with adrenal hemorrhages in TAFRO syndrome.

In addition, the lymphatic anatomy of the adrenal glands is an integral part of their overall structure and function. The adrenal glands have a network of lymphatic vessels responsible for draining lymph from the adrenal glands. The lymphatic network comprises two distinct lymphatic plexuses, one deep in the capsule and the other in the medulla, which is a unique aspect that differentiates adrenal glands from other abdominal organs [30,31]. Lymph from the adrenal glands primarily drains into the lumbar lymph nodes, which are situated along the abdominal aorta. Both the adrenal lymphatic systems and the perirenal lymphatic systems involve the adrenal glands. The perirenal lymphatic system has four major portions, namely, the renal hilar lymphatics, capsular lymphatics, communicating pericapsular lymphatics, and subfascial lymphatics [32]. The renal hilar lymphatics involve adrenal glands [32]. Matsumoto et al. [33] reported that by using a heavily T2-weighted three-dimensional MRI sequence, these lymphatic vessels can be frequently and clearly identified, with the highest detectability in the renal hilar lymphatics, which involve the adrenal glands [32]. These anatomical features may explain the following unexplained characteristics in TAFRO syndrome: first, paraaortic edema and perirenal edema were observed in almost all cases in patients with early stage TAFRO syndrome, even in those without pleural effusion or ascites [7]. This suggests that there is a mechanism by which edema in TAFRO syndrome first develops in the perirenal and paraaortic regions, which are involved in the perirenal and adrenal lymphatic systems [30,32]. Second, adrenal disorders, including adrenal ischemia, adrenal hemorrhage, and adrenomegaly, were frequently observed in patients with TAFRO syndrome, as we reported in previous and present studies [7]. These adrenal abnormalities can be caused by lymphatic congestion. Considering the high affinity of TAFRO syndrome for the lymphatic system, TAFRO syndrome-induced lymphatic congestion may cause these adrenal abnormalities. Furthermore, the anatomical structure in which the lymphatic drainage of the adrenal glands and kidneys joins the aortic lymph drainage towards the cisterna chyli and thoracic duct and the frequency of adrenal pathology, venous, and/or lymphatic congestion may play a marked role in the progression of edema in TAFRO syndrome [7]. To clarify the involvement of the perirenal and adrenal lymphatic systems in TAFRO syndrome, it is necessary to visualize the lymphatic systems. Takahashi et al. [32] introduced several techniques to visualize the lymphatic systems potentially applied for investigating perirenal and adrenal lymphatic systems in patients with TAFRO syndrome: first, delayed contrast-enhanced CT scans (i.e., CT urography); second, CT lymphangiography performed with conventional lymphangiography using an ethiodized oil-based contrast agent or after injecting an iodinated water-soluble contrast agent via the transabdominal access of the cisterna chyli with CT guidance; and third, MR lymphangiography using non-enhanced heavily T2-weighted imaging to depict fluid-filled lymphatic vessels and three-dimensional fat-saturated T1-weighted gradient-echo imaging after the subcutaneous or intranodal injection of gadolinium-based contrast agent. Further studies using these techniques are warranted to verify the hypothesis that perirenal and adrenal lymphatic systems are frequently involved and cause adrenal abnormalities in patients with TAFRO syndrome.

The calcification of the adrenal glands was frequently observed during follow-up (9/14, 64.3%). We consider the calcification of the adrenal glands to be a dystrophic and/or metastatic change related to adrenal damage and renal insufficiency, respectively. Dystrophic changes are indicated because no adrenal calcification was observed through CT early in the disease. However, adrenal calcification appeared on follow-up CT scans in patients with adrenal abnormalities. Kano et al. [15] reported that calcifications in TAFRO syndrome were observed in 46% (6/13) in the adrenal glands, 38% (5/13) in the myocardium, 23% (3/13) in the skeletal muscles, and 15% (2/13) in the gallbladder wall, pancreas, kidneys, and skin, and adrenal calcifications always appeared in follow-up studies in patients with adrenal abnormalities (hemorrhage or adrenomegaly) observed on early CT. This was also the case in our previous study [7] and one additional case in the present study (Figure 2), where patients with bilateral adrenal ischemia/infarction showed adrenal calcifications in follow-up CT scans. In contrast, the metastatic mechanism can also be considered because the calcifications in the adrenal glands and kidneys were always bilateral in the study by Kano et al. [15]. In TAFRO syndrome, renal insufficiency is frequent, and it may facilitate metastatic calcification, as Minomo et al. [34] and Kano et al. [15] suggested.

Since most studies reporting adrenal abnormalities in TAFRO syndrome are case-based, it is difficult to estimate the exact incidence. However, we believe that the incidence of adrenal abnormalities in TAFRO syndrome has been underestimated. In our previous study focusing on TAFRO syndrome with early CT imaging, adrenal ischemia/infarction was observed in five out of thirteen cases (38.5%) [7]. However, the literature search in the present study revealed a few cases of TAFRO syndrome with noted adrenal ischemia/infarction. Given these minor findings, adrenal ischemia/infarction can be easily overlooked, especially when non-radiologists evaluate radiological images. Radiologists familiar with interpreting abdominal imaging are more likely to detect adrenal abnormalities, including size, CT attenuation, and MRI signals, that differ from normal adrenal glands. However, subjective evaluation alone may not be sufficient for interobserver agreement, even among abdominal radiologists. Furthermore, frequent renal insufficiency associated with TAFRO syndrome prevents contrast-enhanced CT, making the diagnosis of adrenal ischemia/infarction more challenging. The incidence of adrenal hemorrhage also seems underestimated. This is partly because adrenal hemorrhage can occur without apparent CT imaging abnormalities. We previously reported a case with autopsy-proven bilateral adrenal hemorrhage [7] despite a CT scan showing only a unilateral adrenal hemorrhage. Adrenal ischemia/infarction and hemorrhage can be detected more easily on MRI. However, further studies with an evaluation of MR images are needed to clarify this. Adrenomegaly was almost always present in patients with adrenal abnormalities, with or without evidence of adrenal ischemia/infarction or hemorrhage. However, adrenomegaly seems to be easily overlooked by subjective evaluation alone; thus, an objective evaluation should be performed. In our previous study [7], we defined adrenomegaly as a 20% or more increase in volume compared to the average adrenal volume of the same age group using whole-organ volumetry. We found that six of the thirteen cases (46.2%) were positive for adrenomegaly. The mechanism of adrenomegaly in TAFRO syndrome is unknown; however, again, it could be due to venous or lymphatic congestion and hemorrhage, as well as part of the anasarca or inflammation associated with TAFRO syndrome.

Additionally, adrenal involvement has been reported in cases of unicentric Castleman’s disease (UCD). Raja et al. [35] summarized cases of adrenal region Castleman’s disease, which were reported to be localized unicentric lesions confined to the adrenal gland and enhanced to varying degrees. However, our literature search did not find a report of adrenal abnormalities associated with non-TAFRO-iMCD. Despite sharing the disease name, UCD and MCD diverge considerably in clinical presentation and prognosis. UCD typically manifests as the proliferation of a single lymph node or a single region of lymph nodes, and patients are usually asymptomatic or present with symptoms related to the mass effect of the lymph node on surrounding structures [36]. Conversely, MCD involves multiple regions of lymph nodes and patients can present with more severe symptoms, which can be fatal [37]. Histologically, the hyaline vascular type accounts for more than 80% of UCD, unlike MCD, which is mostly of the plasma cell type [38]. Although the cause of the lack of evidence of adrenal damage in non-TAFRO-iMCD has been undermined, the difference in the presence of adrenal abnormalities between TAFRO syndrome and non-TAFRO-iMCD may help to differentiate TAFRO syndrome from non-TAFRO-iMCD and supports that TAFRO syndrome and non-TAFRO-iMCD are distinct conditions. To verify this, a prospective study with a sufficient number of patients is desirable to determine the frequency and type of adrenal abnormalities between TAFRO syndrome and non-TAFRO-iMCD patients.

Among the 23 patients with TAFRO syndrome we investigated, abdominal pain was present in 15 patients (65.2%). Notably, all 15 patients (100%) reported abdominal pain from the onset of the disease (Table 1) [7,14,15,16,17,18,19,20,23]. This is statistically significantly higher than the incidence of abdominal pain reported by Iwaki et al. [2] in 25 patients with iMCD-TAFRO (where the presence or absence of adrenal involvement was not specified; 15/23 vs. 8/25, *p* < 0.05 for Fisher’s exact test). This result agrees with Nishimura et al. [39], who reported that the incidences of abdominal pain were 50.0% and 28.6% in TAFRO syndrome and non-TAFRO-iMCD, respectively. As Fujimi et al. [14] emphasized, adrenal abnormalities should be carefully evaluated in TAFRO syndrome when abdominal pain is present at the onset of the disease. Furthermore, TAFRO syndrome should be included in the differentials of acute abdomen.

This study has some limitations. First, due to the rarity of TAFRO syndrome, the number of cases with abnormalities was limited. However, as already mentioned, adrenal abnormalities can be easily overlooked on CT and MRI, especially when evaluated by non-radiologists, and the detectability of adrenomegaly is reduced without objective evaluation by whole-organ volumetry. Second, the exact incidence of adrenal abnormalities in TAFRO syndrome is unknown because most of the included studies were case-based. Third, the mechanisms of adrenal abnormalities and the histological investigation on the affected adrenal glands were undermined. Fourth, because most of the included studies were case-based, selection bias might have been introduced. Future prospective studies with a larger number of patients, radiological investigations by radiologists, and histological investigations of affected adrenal glands are warranted to validate our findings and improve generalizability. Collaborative research using an international registry of TAFRO syndrome will be necessary to better understand this potentially heterogeneous syndrome.

## 5. Conclusions

This article reviewed the clinical and radiological findings of patients with adrenal abnormalities in TAFRO syndrome. Adrenal ischemia/infarction, adrenal hemorrhage, and adrenomegaly have been reported in patients with TAFRO syndrome, and most of these patients have experienced abdominal pain since the disease’s onset. Since adrenal abnormalities have rarely been reported in non-TAFRO-iMCD patients, they may serve as differential diagnostic markers between TAFRO syndrome and non-TAFRO-iMCD. The early detection of adrenal abnormalities may lead to earlier diagnoses and the better management of TAFRO syndrome.

## Data Availability

The data presented in this study are available on reasonable request from the corresponding author.
